# SETDB1 amplification in osteosarcomas: Insights from its role in healthy tissues and other cancer types

**DOI:** 10.18632/oncotarget.28688

**Published:** 2025-02-12

**Authors:** Elodie Verdier, Nathalie Gaspar, Maria Eugenia Marques Da Costa, Antonin Marchais

**Affiliations:** ^1^UMR 1015 Tumour Immunology and anti-cancer immunotherapy Unit, Gustave Roussy Cancer Campus, Villejuif 94800, France; ^2^Department of Oncology for Child and Adolescent, Gustave Roussy Cancer Campus, Université Paris-Saclay, Villejuif 94805, France

**Keywords:** SETDB1, cancer epigenetics, tumor immunogenicity, mesenchymal differentiation in osteosarcoma

## Abstract

Epigenetic modifications, which reversibly regulate gene expression without altering the DNA sequence, are increasingly described in the literature as essential elements in the processes leading to cancer development. SETDB1 regulates histone 3 (H3) K9 di- and trimethylation, promoting heterochromatin formation, and plays a key role in gene silencing. Epigenetic deregulation of *SETDB1* expression appears to be involved in different cancers types, particularly in aggressive, relapsing or treatment-resistant subtypes. Despite advances in research, the full range of mechanisms through which this protein acts remains unclear; however, it is evident that SETDB1 has a pivotal role, particularly in the mesenchymal stem cells differentiation, tumor evasion and treatment resistance. Its role in genetically complex sarcomas, such as osteosarcoma, has not been fully explored, although recent Omics analyses suggest its presence and amplification in osteosarcoma. Given its involvement in osteoblastogenesis and adipogenesis, we discuss the potential of SETDB1 as a key target for new therapeutic strategies in osteosarcoma.

## INTRODUCTION

Advances in biotechnology and genetics have provided a deeper understanding of the various mechanisms that can lead to oncogenesis and tumor aggressiveness. However, some tumor types, such as sarcomas, remain poorly understood and involve complex genetic processes, including multiple rearrangements and few recurrent somatic mutations. These complexities are often associated with limited or no therapeutic options [1]. Researchers are investigating the involvement of microenvironment, intercellular signals, immune system interactions, and global homeostasis in tumorigenesis. Tumor cells can manipulate and control these elements to promote their proliferation and dissemination [2–5]. For instance, recent studies have shown, that tumors can modulate neuroendocrine secretions to create a more favorable environment for their growth [6]. Sarcomas are a heterogeneous group of tumors of mesenchymal origin, accounting for about 15% of all cancers in children and young adults. Among sarcomas, osteosarcoma is the most prevalent malignant bone tumor in adolescents and young adults [7]. It is an aggressive tumor, and its current treatment consists of multidrug chemotherapy and surgical resection. Despite extensive efforts to understand the complex genetics of osteosarcoma or its microenvironment, no new, effective, therapeutic strategies have emerged [8, 9]. The survival rate remained stagnant over the past few decades (70% for localized forms and less than 30% for metastatic or treatment-resistant forms at diagnosis) due to chemotherapy resistance and metastatic recurrence.

Several recent studies on the biology and genetics of sarcomas, including osteosarcoma, have highlighted the role of epigenetics as a key component of tumor cell plasticity and the regulation of neoantigen expression [1, 10]. Numerous genes involved in epigenetic regulation have been identified, which are capable of controlling transcription and, consequently, cell fate at various levels, particularly in mesenchymal tumors [11]. The definition of epigenetics encompasses DNA modifications such as acetylation, methylation, phosphorylation, ubiquitination, and sumoylation, which reversibly regulate gene expression. DNA methylation catalyzes chromatin changes leading to heterochromatin formation. Two distinct groups of proteins are involved: the PRMT1 family and the SET domain-containing protein family (which affect arginine and lysine residues) [12, 13].

Whole exome sequencing of osteosarcoma samples from both diagnosis and relapses has highlighted several factors, including *SETDB1*, that are amplified in the most aggressive forms of the disease [14]. In addition, current analyses show a correlation between *SETDB1* presence, DNA methylation levels of epigenomic targets, tumor aggressiveness, and response to treatments [15]. In this work, we will discuss the role of SETDB1 in the healthy state, in mesenchymal differentiation, and how its amplification may contribute to the plasticity and immune escape of osteosarcoma cells.

After reviewing the literature on the complex genetics of sarcomas, with a particular focus on osteosarcoma, we shifted our attention to the mechanisms of epigenetic regulation. In this review, only articles specifically describing SETDB1 were retained, sourced from bibliographic databases. The following keywords (SETDB1 osteosarcoma, epigenetic regulation in sarcoma, EMT, mesenchymal stem cells, immune escape, etc.) allowed us to categorize the articles, first focusing on the description of the protein itself, and then expanding to its role in the various related domains. Articles that did not specifically mention SETDB1 were excluded.

## SETDB1: STRUCTURE AND INTERACTIONS

The “SET domain-bifurcated histone lysine methyltransferase 1” (SETDB1, also known as ESET, “ERG-associated protein with SET domain”) catalyzes di- and tri-methylation of histone H3 at lysine 9 (H3-K9), resulting in two distinct histone marks: H3K9me2 and H3K9me3 [16–18]. This modification leads to heterochromatin formation, by creating a binding site for heterochromatin protein 1 (HP1), with the primary consequence of gene expression silencing. These essential epigenetic modifications play a key role in the repression of satellite repeats and transposable elements [19, 20].

**Figure 1:**
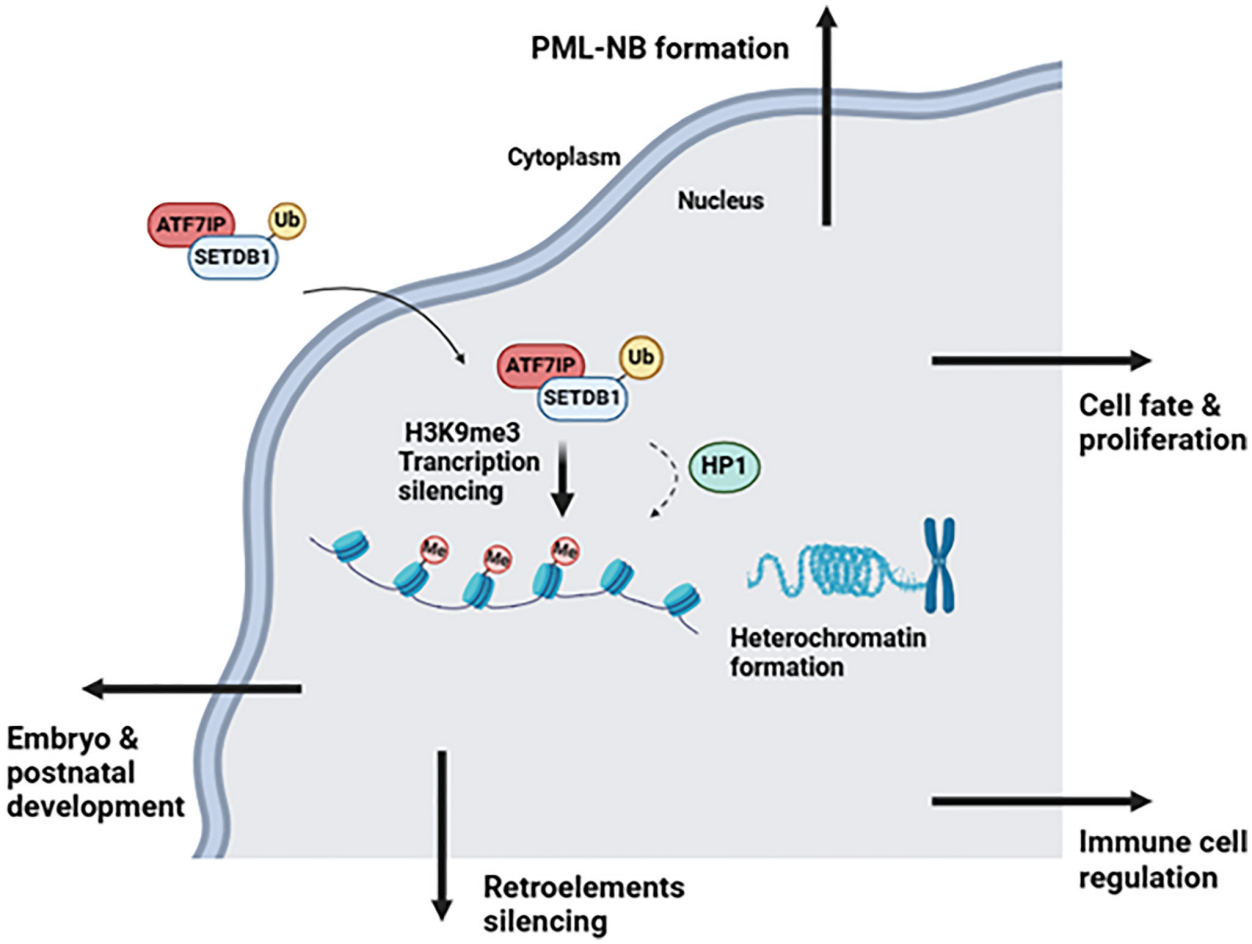
SETDB1 main functions, generated with BioRender (http://biorender.com).

The main known and described functions of SETDB1 include embryonic and postnatal development, promyelocytic leukemia nuclear body (PML-NB) formation, retroelement silencing, modulation of cell fate and proliferation, and immune cell regulation (Figure 1).

**Figure 2:**
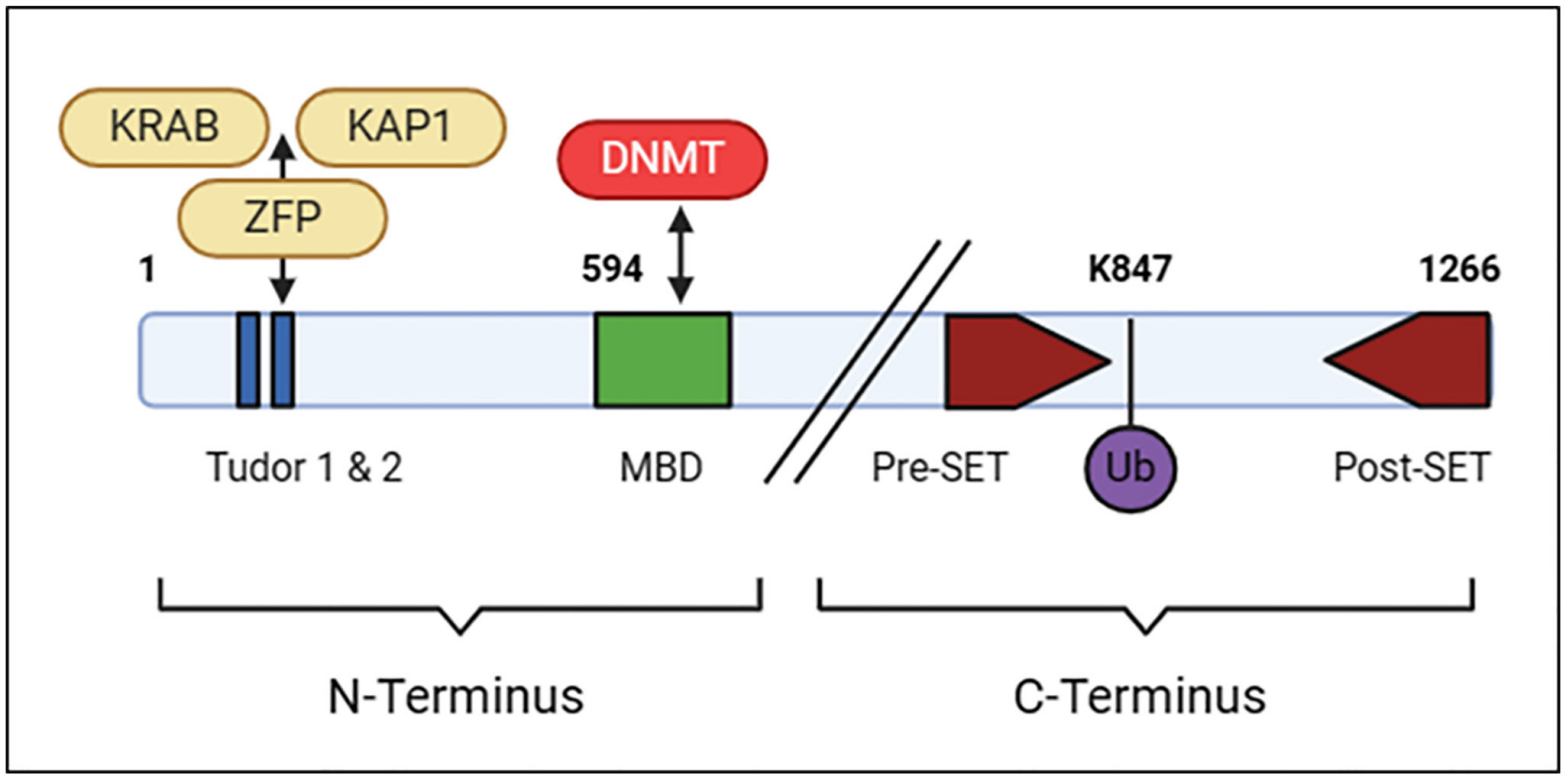
SETDB1 protein structure generated with BioRender (http://biorender.com).

The SETDB1 protein structure, which includes three isoforms generated through alternative splicing, features a bifurcated SET domain and conserved amino acids found in other species, forming an interacting chain. The C-terminus of SETDB1 is responsible for the methylation reaction, while the N-terminus interacts with chromatin modification enzymes, such as DNA methyltransferases, particularly DNMT3, through its methyl-CpG-binding domain (MBD), leading to trimethylation of H3K9. Two Tudor domains located in the N-terminus facilitate the formation of complexes with other regulatory factors (Figure 2) [21, 22].

H3K9me3, generated by SETDB1, interacts with ATF7IP (activating transcription factor 7-interacting protein 1), a cofactor that facilitates the recruitment of HP1. The resulting complex plays a key role in forming heterochromatin by altering the spatial conformation of euchromatin from an “open” to a “closed” state, which restricts gene accessibility. Consequently, SETDB1 has a mostly suppressive role on gene expression [23], affecting indirectly other histone modifications, through mechanisms that remain poorly understood [21].

Beyond its activity on H3K9me3, SETDB1 also regulates cell fate and cell lineage by controlling the association of Cohesin with unique topological domains, recently identified as DiSCs (domains involving SETDB1 and Cohesin). Within the genome, this system represents a non-canonical model for SETDB1 binding [24].

Under physiological conditions, particularly during embryonic differentiation and development of the central nervous system (CNS), SETDB1 plays a key role [25]. It is also essential for maintaining X chromosome silencing in female mammalian cells [26]. Loss of H3K9me3 and dysregulation of SETDB1 have been associated with aging, neurological disorders, obesity, altered tissue integrity, and, ultimately, carcinogenesis [27–29].

## SETDB1 ROLE IN DIFFERENTIATION OF MESENCHYMAL STEM CELLS

During embryogenesis and postnatal development, SETDB1 regulates cell stemness and cell fate by controlling the differentiation of MSCs through the modulation of transcription factors. During embryonic skeletal development, SETDB1/ESET, as a repressor, regulates the transactivating ability of RUNX2 (a hypertrophy-promoting transcription factor) indispensable for osteoblast differentiation [30]. SETDB1 can bind to and inhibit RUNX2 activity through its association with histone deacetylase 4, forming a multi-protein complex that activates SETDB1 intrinsic methyltransferase activity. This suppresses RUNX2-mediated transactivation of the osteocalcin gene [31]. Mesenchymal stem cells lose their ability to differentiate into osteoblasts in *ESET* knockout, which instead promotes the hypertrophic differentiation and apoptosis of articular chondrocytes. This is explained by the upregulation of matrix metalloproteinases (MMPs) and disintegrin and metalloproteinase with thrombospondin motifs (ADAMTs), enzymes responsible for matrix degradation and typically regulated by RUNX2 [30]. In addition to the disorganization of growth plate chondrocytes, *SETDB1* seems to regulate epiphyseal plate formation and, when deleted, disrupts long bone growth [31, 32]. In the same way, through its action on the transcription factor peroxisome proliferator-activated receptor gamma PPARƔ - an inhibitor of osteoblastogenesis and inducer of adipogenesis - *SETDB1* regulates adipogenic differentiation of MSCs [33]. Two signaling pathways coexist: the canonical Wnt/beta-catenin pathway, which inhibits PPARƔ mRNA expression and the noncanonical Wnt pathway, which activates SETDB1, leading to PPARƔ repression via histone H3K9 methylation of its target genes [34]. In addition, another cellular pathway involving SETDB1, but independent of its methyltransferase activity, seems to repress adipogenic genes and inhibit pre-adipocyte differentiation in an independent DNA methylation way. Researchers suggest that SETDB1 ubiquitination, necessary for its activity, is not fully present during gene repression, implying the existence of an alternative pathway [35].

SETDB1, by inhibiting the expression of the endogenous retroviruses (ERVs) family of transposable elements, plays a crucial role in maintaining muscle tissue integrity and facilitating repair following injury. ERVs are known to trigger muscle cell death after once these cells exit their quiescent state. *In vivo*, in muscle stem cells (MuSCs), *SETDB1* absence leads to an uncontrolled inflammatory response mediated by the interferon pathway and cytokine release, ultimately causing necrosis [36]. This pro-inflammatory response occurs through the activation of the DNA-sensing cGAS-STING pathway. These two SETDB1-regulated events, are essential to maintaining a delicate balance between tissue regeneration and inflammatory response.

SETDB1 appears to be a key regulator of mesenchymal stem cell fate, influencing both adipogenesis and osteoblastogenesis. Considering the pathogenicity of osteosarcoma and its MSC-related origin, this protein could play a pivotal factor in tumor proliferation.

## SETDB1 IN TUMORIGENESIS

During oncogenesis, *SETDB1* transcription is significantly upregulated, associated with tumorigenesis promotion and modulation of genes involved in various oncologic signaling pathways. *SETDB1* gain-of-function mutations promote proliferation, invasion and migration of cancer cells [37–39].

Depending on the cellular context, *SETDB1* can act either as a proto-oncogene, by silencing tumor suppressor genes such as APOE, p53 and HoxA, or as a tumor suppressor through the downregulation of the oncogene ANXA2 [18]. For example, under severe hypoxic conditions, *SETDB1* regulates p53-induced apoptosis [40], whereas in others conditions, it promotes tumorigenesis by increasing the expression of cancer-related genes. *SETDB1* is involved in multiple signaling pathways, including WNT, focal adhesion, MAPK, insulin, Toll-like receptors (TLR), and JAK-STAT pathways [29]. Additionally, *SETDB1* can promote AKT1 signaling and repress pro-apoptotic genes transcription. Given that AKT1 hyperactivation is associated with poor prognosis in several cancers, this role of *SETDB1* further underscores its importance in cancer progression [23].

Epigenetic deregulation of *SETDB1* expression seems to be involved in various types of cancers, including melanoma [37], hepatocellular carcinoma, ovarian [41], lung cancer, colorectal [42] and breast cancer [29, 43]. *SETDB1* is notably overexpressed in aggressive types of diseases and is often implicated in the epigenetic regulation of tumor progression and metastasis. In breast tumors, *SETDB1* plays a significant role in promoting metastasis by facilitating the acquisition of stem-cell-like properties and activating EMT programs [23]. *In vitro*, loss of *SETDB1* blocks cell invasion and migration, resulting *in vivo* into a reduction of lung metastasis. On the contrary, through a direct binding to the promoter of the transcription factor *SNAIL1,* overexpression of *SETDB1* enhances cell invasiveness by acting as an EMT inducer [44]. In the same way, *SETDB1* downregulates MiR7 leading to *STAT3* suppression, which inhibits BCSCs metastasis *in vivo* [45]. It also suppresses *FOXA2* expression, a key metastasis regulator, promoting NSCLC (non-small cell lung cancer) cells invasion and migration [39]. In ovarian cancer cells, knockdown of *SETBD1* prevents cells migration and motility by regulating *SF3B4* expression and influencing the tumor immune microenvironment [41]. In colorectal cancer, *SETDB1* drives tumor development and proliferation by downregulating the tumor suppressor factor p21 and promoting EMT regulation [42]. In myeloma cells, treatment resistance has been associated with the role of *SETDB1* in PI3K/AKT pathway and its involvement in epithelial-mesenchymal transition processes [46].

In pediatric high-grade gliomas (pHGG), gene silencing of *SETDB1* leads to a significant reduction in cell viability and proliferation, while promoting apoptosis. *SETDB1* silencing also reduces the expression of mesenchymal markers and decreases migration capacity of pHGG cells. Analysis of EMT markers following *SETDB1* silencing revealed a downregulation of CDH2, the MARCKS gene, and reduced Snail levels [47]. At the opposite, experiments on lung cancer cells have shown that inhibition of *SETDB1* using the CRISPR/Cas9 system decreases proliferation capacity but unexpectedly increases migration and transformation activities. In *SETDB1* knock out studies, researchers observed downregulation of B-catenin and E-cadherin expression, modifications in E-cadherin cellular localization, and increased levels of STAT3 and Akt [48].

To date, very few studies describe the role of SETDB1 in sarcomas, and in osteosarcoma specifically, despite genetic analyses suggesting its involvement in osteosarcoma pathophysiology [14]. Through the epigenetic regulation of *GRIK2*, a known tumor-suppressor gene identified in gastric cancer, *SETDB1* appears to enhance cell proliferation, migration, and apoptosis resistance in osteosarcoma [49, 50].

## SETDB1 AND IMMUNE PATHWAYS

*SETDB1* appears to suppress tumor-intrinsic immunogenicity. One of the most prominent regulation of immune response mediated by *SETDB1* is its ability to prevent tumor cells from evading innate immune sensing by limiting endogenous retrotransposon expression. The first report of this function in cancer cells was published by Cuellar TL et al. in 2017 when the authors demonstrated that *SETDB1* silencing, by decreasing H3K9me3 at repetitive loci in AML cells, elevates the expression of IFN-β and interferon-stimulated genes [51]. More recently, Johnson et al. provided further insight into the diverse mechanisms by which *SETDB1* regulates the immune system, particularly in tumors. Specifically, *SETDB1* is involved in the methylation of the promoter regions of interleukins 2 and 7, modulates T cell function and development, and interferes with B lineage differentiation through endogenous retroviruses (ERVs) repression [52]. Human ERVs, derived from ancestral infections, account for approximately 8% of our genetic heritage and play a pathogenic role in immune diseases [53–55]. Beyond cancer, SETDB1 appears to be involved in immune modulation observed in autoimmune diseases such as immune thrombocytopenia (ITP), where the transcriptional levels of human ERVs correlate with those of *TRIM28/SETDB1* [56]. Similar to KDM5B, an H3K4 demethylase, *SETDB1* can function as an epigenetic checkpoint, preventing the presentation of antigenic determinants derived from transposable elements (TEs). KDM5B recruits SETDB1 to induce epigenetic silencing and ensures its retention in the cell nucleus. In invasive melanoma cells, this mechanism interferes with cancer stem cell-targeting responses by limiting the exposure of antigenic determinants, immunostimulatory cytokines secretion and other pro-inflammatory signals [57, 58]. When *SETDB1* is lost, the repression of transposable elements is disrupted, enabling the production of major histocompatibility complex class I (MHC-I) peptides, the encoding of viral proteins, and the triggering of T-cell responses. Similarly, in human tumors, immune exclusion and resistance to checkpoint blockade are associated with *SETDB1* amplification [58]. Overall, the deletion of *SETDB1* appears to enhance antitumor immune responses: antigen expression and presentation are increased, and T-cell activation is stimulated. Additionally, SETDB1 deficiency has been linked to impaired B-cell development [59].

Furthermore, during T-cell maturation, selection, and lineage development, SETDB1 influences various intracellular signaling genes through its H3K9me3 activity. Upon TCR stimulation, SETDB1 silencing in thymocytes results in impaired ERK activation, which is partially explained by the ectopic expression of the inhibitor FcγRIIB [60].

All these recent discoveries suggest that a balance must be achieved in SETDB1 expression: reducing its pro-tumoral action while preserving its pro-immunity function. Furthermore, three major repressive complexes-KRAB-ZFP, HUSH and KAP1/TRIM28-have been recently identified and shown to mediate immune modulation by SETDB1*,* depending on the cellular context. HUSH can recruit SETDB1 to silence genes, KRAB-ZFP activates H3K9me3 by binding to specific DNA motifs, and TRIM28, as a major corepressor protein, can bind to the KRAB domain and recruit *SETDB1*. In leukocytes, ERV repression is modulated by the SETDB1-KAP1 complex, whereas in melanocytes, KDM5B recruits SETDB1 [52].

How SETDB1 influences the tumor immunogenicity of osteosarcoma remains to be further investigated. In osteosarcoma, the tumor microenvironment-and particularly its immune infiltration-appears to be predominantly composed of T cells and macrophages [61, 62]. While immunotherapy has proven effective in certain types of cancer, it has not yet shown significant benefits in osteosarcoma. For instance, the pro-tumoral role of interferons (IFN) described in osteosarcoma did not translate into clinical benefit for patients treated with IFN-α2b maintenance therapy, as demonstrated in the EURAMOS-1 clinical trial [63]. Given SETDB1’s role in T cell regulation, it could represent a promising therapeutic target. Its dual role in preventing antigenic expression and promoting immune exclusion warrants further investigation in osteosarcoma, a cancer which, despite its abundant chromosomal rearrangements, fails to express antigens.

## SETDB1 AS A THERAPEUTIC TARGET

The current standard treatment for osteosarcoma consists of chemotherapy and surgery. Unfortunately, little progress has been made over the past forty years in improving survival rates for refractory or inoperable patients. Despite advancements in modern biology, no specific therapeutic targets have been identified, highlighting the need to investigate the role of protein such as SETDB1. New therapies under investigation include anti-PD1 therapies, inhibitors of the VEGF (vascular endothelial growth factor) and PDGF (platelet-derived growth factor) pathway, PI3K/mTOR pathway inhibitors, MYC oncogene inhibitors, and IGF (insulin-like growth factor) inhibitors. Although some current studies show promising results , their efficacy remains imperfect [8].

Until now, no specific inhibitors have been used in SETDB1 studies. Instead, 3’-deazaneplanocin A (DZNep), paclitaxel, mithramycin A, and microRNA therapeutics have been primarily explored as inhibitory strategies targeting SETDB1 [23, 29, 45, 64, 65].

In lung cancer cells, the non-specific inhibitor DZNep targets various HMTases, reduces their expression levels, and promotes apoptosis. However, its principal limitation is its lack of specificity [23, 66].

Paclitaxel, an anticancer molecule produced by endophytic fungi, downregulates SETDB1 expression by binding to its promoter region and also enhances p53 expression [64].

MicroRNA therapeutics represent promising candidates for replacing or enhancing the activity of underexpressed tumor-suppressor microRNAs in tumor cells. For example, as proposed by Zhang et al*.* [45], miR-7 might be introduced to control tumorigenesis via SETDB1 regulation. However, to date, these therapeutic molecules have shown limited clinical benefit.

Mithramycin A, an antitumor antibiotic, is a transcription factor inhibitor that recognizes and binds to GC-rich promoter regions of oncogenic genes, such as *SETDB1*. A study demonstrated that mithramycin A has significant effects on H3K9me3 signatures and *SETDB1* expression [65]. Although mithramycin A exhibits potent anticancer activity, it is associated with severe side effects due to its non-specific action. To overcome this limitation, combinatorial biosynthesis has led to the development of several mithramycin analogs, referred to as “Mithralogs”, which demonstrate superior antitumor efficacy an improved toxicity profile [29].

Recently, a selective cell-active inhibitor of *SETDB1* Tudor Domain was discovered. SETDB1-TTD-IN-1 TFA is a potent, competitive and selective small-molecule inhibitor that targets the *SETDB1* tandem Tudor domain (SETDB1-TTD) [67]. By competing with endogenous binders, this molecule disrupts the interaction of SETDB1-TTD with methylated lysine residues, thereby blocking its recognition. This discovery offers a valuable tool for scientists to better understand the precise biological functions of SETDB1-TTD. All these molecules are summarized in Table 1.

**Table 1:** SETDB1 inhibitor molecules

Inhibitors	Action	Limitations
**DZNep**	**Epigenetic regulation** Inhibites S-adenosyl homocystein hydrolase: H3K27me3 inhibitor	No specific and limited action
**Paclitaxel**	**Cell cycle** Chemotherapy inhibiting tubulin depolymerization **Epigenetic regulation** Down-regulates *SETDB1* gene expression in a p53 dependent manner	No specific action; Unknown epigenetic mechanism of action
**MiR-7**	**Gene translation blocking** Directly targets the 3’UTR of SETDB1	Fragile, unstable molecules, requiring a vector for action
**Mithramycin A**	**Transcriptional regulation** binding actions and competes for GC-rich promoter regions of SETDB1	Limited clinical activity due to tumor heterogeneity
**SETDB1-TTD-IN-1 TFA**	Selective endogenous binder-competitive small-molecule SETDB1 tandem tudor domain (TTD) inhibitor	Fundamental research only, under study

It is well established that SETDB1 regulates gene expression by interacting with multiple factors. Therefore, targeting downstream proteins regulated by SETDB1 may offer a strategy to increase the specificity of therapeutic molecules. For example, SETDB1 modulates the transcriptional activity of PPARγ, a lipid-binding nuclear receptor involved in osteoblastogenesis mechanisms. PPARγ is targetable by T0070907, an available irreversible inverse agonist, which has shown potential interest in osteosarcoma (OS) progression [68–70].

Despite the promise raised by such specific inhibitors, studies on similar types of molecules have yet to demonstrate clear clinical benefits and thus require further investigation.

The identification of a SETDB1 inhibitor could provide new hope for the treatment of osteosarcoma by modulating its anti-tumor immune activity and altering its microenvironment, potentially enhancing tumor sensitivity to therapeutic approaches, such as radiotherapy.

## SETDB1 AND RADIO-SENSITIVITY

In osteosarcoma, radiotherapy is typically used for unresectable disease to achieve better local control. However, in most cases, this treatment is ineffective due to unknown mechanisms. Osteosarcoma often requires high doses of radiation to achieve disease control [71, 72]. Cellular exposure to ionizing radiation can activate the type I interferon response, which plays a crucial role in tumor response to radiotherapy through the cGAS/STING signaling pathway. DNA methyltransferase inhibitors have been shown to promote ERV activation, leading to the production of type I interferons by inducing a viral mimicry state. This is achieved through the generation of cytoplasmic double-stranded RNAs (dsRNA) and the activation of the RIG-I–MDA5-MAVS signaling pathway [73, 74]. SETDB1 plays a crucial role in suppressing ERV activation by maintaining heterochromatin. Radiation can promote ERV activation by significantly attenuating *SETDB1* expression, which leads to a downregulation of H3K9 trimethylation. Furthermore, *SETDB1* loss could significantly enhance ERV activation and type I interferon production, thereby sensitizing murine tumors to radiotherapy, which is dependent on cytotoxic T cells and type I interferons [59]. Tumor radiosensitivity appears to be inversely correlated with *SETDB1* expression, through mechanisms that influence cellular immune infiltration, including T cells, macrophages, NK cells, and dendritic cells [52, 75]. These findings are consistent with its role in immune escape and the regulation of various pro-inflammatory actors involved in therapeutic responses in oncology, particularly in osteosarcoma.

## SETDB1 AMPLIFICATION MIGHT PLAY A MAJOR ROLE IN OSTEOSARCOMA TREATMENT AND IMMUNE ESCAPE

Altogether, studies on SETDB1 paint the picture of a key protein involved in tumor progression, resistance to therapeutics and MSC commitment [18, 23, 29, 44], although its role in osteosarcoma remains poorly characterized [49, 50]. *SETDB1* amplification is associated with increased aggressiveness and therapeutic tolerance through several mechanisms, particularly in tumor immunogenicity [51, 57, 58]. *SETDB1* expression, through the mechanisms described above, influences the response to anti-cancer therapies, including radiotherapy [52, 59, 75].

**Figure 3:**
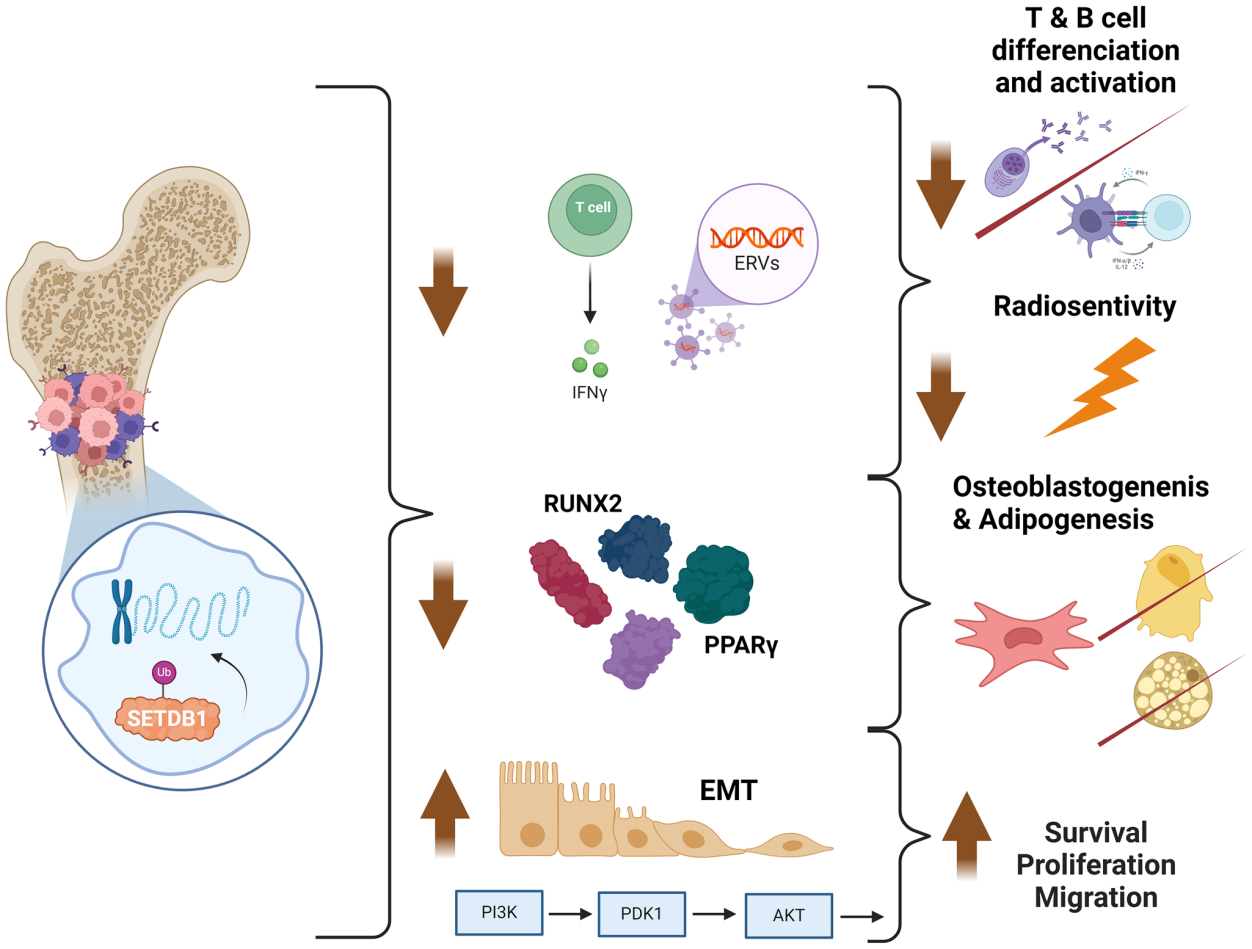
Proposed summary of the role of SETDB1 in osteosarcoma (http://biorender.com).

In a recent study, we confirmed, through whole exome sequencing of osteosarcoma samples from diagnosis and relapses, as well as their derived PDX models, the presence of *SETDB1* amplification, predominantly in relapses [14]. Given its involvement in osteoblastogenesis and adipogenesis [30–32], this protein could play a significant role in the formation and proliferation of osteosarcomas, as well as in promoting migration and metastasis (Figure 3).

The challenge of specifically targeting this protein is twofold: to block its action on MSC differentiation and to reinstate the cytotoxic activity of the immune system. The recent discovery of a specific inhibitor, SETDB1-TTD-IN-1 TFA, provides a valuable opportunity to study SETDB1’s action on tumor cells.

As mentioned above, there are few, if any, studies describing SETDB1 in osteosarcoma, and we are only beginning to understand how this protein can shape the expression of downstream targets.

## CONCLUSIONS AND FUTURE PERSPECTIVE

Osteosarcoma is characterized by a complex genetic profile that leads to significant genetic instability, which contributes to therapeutic resistance. This is one of the reasons why there have been no new therapeutic breakthroughs in recent decades, and survival rates remain stable, with the persistent problem of refractory or relapsed disease. Technological advances have enabled a detailed description of the genetic landscape of tumors, thus thereby deepening our understanding of the origins of tumorigenesis. A stratification of osteosarcomas has been developed based on these methods, opening up new and, better-targeted therapeutic avenues [76].

In recent years, epigenetics has emerged as a key mechanism in oncogenesis and cancer aggressiveness. SETDB1*,* through its role in histone methylation, is a major player in heterochromatin formation, and thus promotes or prevents the expression of numerous genes linked to carcinogenesis. Its involvement in various cancers has been well studied, but its role in osteosarcoma remains unclear. Our team has demonstrated the presence of *SETDB1* abnormalities, particularly its amplification, through WES analysis of osteosarcoma human samples and cell lines [14]. Therefore, it is now essential to continue research on the role of *SETDB1* in osteosarcoma and investigate whether its inhibition could provide a pathway to improve patient care.

## References

[1] Nacev BA , Jones KB , Intlekofer AM , Yu JSE , Allis CD , Tap WD , Ladanyi M , Nielsen TO . The epigenomics of sarcoma. Nat Rev Cancer. 2020; 20:608–23. 10.1038/s41568-020-0288-4. 32782366 PMC8380451

[2] Bakhshandeh S , Werner C , Fratzl P , Cipitria A . Microenvironment-mediated cancer dormancy: Insights from metastability theory. Proc Natl Acad Sci U S A. 2022; 119:e2111046118. 10.1073/pnas.2111046118. 34949715 PMC8740765

[3] Risom T , Glass DR , Averbukh I , Liu CC , Baranski A , Kagel A , McCaffrey EF , Greenwald NF , Rivero-Gutiérrez B , Strand SH , Varma S , Kong A , Keren L , et al. Transition to invasive breast cancer is associated with progressive changes in the structure and composition of tumor stroma. Cell. 2022; 185:299–10.e18. 10.1016/j.cell.2021.12.023. 35063072 PMC8792442

[4] Lambert AW , Pattabiraman DR , Weinberg RA . Emerging Biological Principles of Metastasis. Cell. 2017; 168:670–91. 10.1016/j.cell.2016.11.037. 28187288 PMC5308465

[5] Francis N , Borniger JC . Cancer as a homeostatic challenge: the role of the hypothalamus. Trends Neurosci. 2021; 44:903–14. 10.1016/j.tins.2021.08.008. 34561122 PMC9901368

[6] Slominski RM , Raman C , Chen JY , Slominski AT . How cancer hijacks the body’s homeostasis through the neuroendocrine system. Trends Neurosci. 2023; 46:263–75. 10.1016/j.tins.2023.01.003. 36803800 PMC10038913

[7] Mirabello L , Troisi RJ , Savage SA . International osteosarcoma incidence patterns in children and adolescents, middle ages and elderly persons. Int J Cancer. 2009; 125:229–34. 10.1002/ijc.24320. 19330840 PMC3048853

[8] Hu Z , Wen S , Huo Z , Wang Q , Zhao J , Wang Z , Chen Y , Zhang L , Zhou F , Guo Z , Liu H , Zhou S . Current Status and Prospects of Targeted Therapy for Osteosarcoma. Cells. 2022; 11:3507. 10.3390/cells11213507. 36359903 PMC9653755

[9] Sayles LC , Breese MR , Koehne AL , Leung SG , Lee AG , Liu HY , Spillinger A , Shah AT , Tanasa B , Straessler K , Hazard FK , Spunt SL , Marina N , et al. Genome-Informed Targeted Therapy for Osteosarcoma. Cancer Discov. 2019; 9:46–63. 10.1158/2159-8290.CD-17-1152. 30266815 PMC7134333

[10] Kresse SH , Rydbeck H , Skårn M , Namløs HM , Barragan-Polania AH , Cleton-Jansen AM , Serra M , Liestøl K , Hogendoorn PC , Hovig E , Myklebost O , Meza-Zepeda LA . Integrative analysis reveals relationships of genetic and epigenetic alterations in osteosarcoma. PLoS One. 2012; 7:e48262. 10.1371/journal.pone.0048262. 23144859 PMC3492335

[11] Grand’Maison A , Kohrn R , Omole E , Shah M , Fiorica P , Sims J , Ohm JE . Genetic and environmental reprogramming of the sarcoma epigenome. Adv Pharmacol. 2023; 96:283–17. 10.1016/bs.apha.2022.10.001. 36858777

[12] Zhang Y , Reinberg D . Transcription regulation by histone methylation: interplay between different covalent modifications of the core histone tails. Genes Dev. 2001; 15:2343–60. 10.1101/gad.927301. 11562345

[13] Lachner M , Jenuwein T . The many faces of histone lysine methylation. Curr Opin Cell Biol. 2002; 14:286–98. 10.1016/s0955-0674(02)00335-6. 12067650

[14] da Costa MEM , Droit R , Khneisser P , Gomez-Brouchet A , Adam-de-Beaumais T , Nolla M , Signolles N , Torrejon J , Lombard B , Loew D , Ayrault O , Scoazec JY , Geoerger B , et al. Longitudinal characterization of primary osteosarcoma and derived subcutaneous and orthotopic relapsed patient-derived xenograft models. Front Oncol. 2023; 13:1166063. 10.3389/fonc.2023.1166063. 37377921 PMC10291137

[15] Vural S , Palmisano A , Reinhold WC , Pommier Y , Teicher BA , Krushkal J . Association of expression of epigenetic molecular factors with DNA methylation and sensitivity to chemotherapeutic agents in cancer cell lines. Clin Epigenetics. 2021; 13:49. 10.1186/s13148-021-01026-4. 33676569 PMC7936435

[16] Wang H , An W , Cao R , Xia L , Erdjument-Bromage H , Chatton B , Tempst P , Roeder RG , Zhang Y . mAM facilitates conversion by ESET of dimethyl to trimethyl lysine 9 of histone H3 to cause transcriptional repression. Mol Cell. 2003; 12:475–87. 10.1016/j.molcel.2003.08.007. 14536086

[17] Cruz-Tapias P , Robin P , Pontis J , Maestro LD , Ait-Si-Ali S . The H3K9 Methylation Writer SETDB1 and its Reader MPP8 Cooperate to Silence Satellite DNA Repeats in Mouse Embryonic Stem Cells. Genes (Basel). 2019; 10:750. 10.3390/genes10100750. 31557926 PMC6826936

[18] Torrano J , Al Emran A , Hammerlindl H , Schaider H . Emerging roles of H3K9me3, SETDB1 and SETDB2 in therapy-induced cellular reprogramming. Clin Epigenetics. 2019; 11:43. 10.1186/s13148-019-0644-y. 30850015 PMC6408861

[19] Delaney CE , Methot SP , Kalck V , Seebacher J , Hess D , Gasser SM , Padeken J . SETDB1-like MET-2 promotes transcriptional silencing and development independently of its H3K9me-associated catalytic activity. Nat Struct Mol Biol. 2022; 29:85–96. 10.1038/s41594-021-00712-4. 35102319 PMC8850192

[20] Dodge JE , Kang YK , Beppu H , Lei H , Li E . Histone H3-K9 methyltransferase ESET is essential for early development. Mol Cell Biol. 2004; 24:2478–86. 10.1128/MCB.24.6.2478-2486.2004. 14993285 PMC355869

[21] Strepkos D , Markouli M , Klonou A , Papavassiliou AG , Piperi C . Histone Methyltransferase SETDB1: A Common Denominator of Tumorigenesis with Therapeutic Potential. Cancer Res. 2021; 81:525–34. 10.1158/0008-5472.CAN-20-2906. 33115801

[22] Lazaro-Camp VJ , Salari K , Meng X , Yang S . SETDB1 in cancer: overexpression and its therapeutic implications. Am J Cancer Res. 2021; 11:1803–27. 34094655 PMC8167684

[23] Batham J , Lim PS , Rao S . SETDB-1: A Potential Epigenetic Regulator in Breast Cancer Metastasis. Cancers (Basel). 2019; 11:1143. 10.3390/cancers11081143. 31405032 PMC6721492

[24] Warrier T , El Farran C , Zeng Y , Ho BSQ , Bao Q , Zheng ZH , Bi X , Ng HH , Ong DST , Chu JJH , Sanyal A , Fullwood MJ , Collins JJ , et al. SETDB1 acts as a topological accessory to Cohesin via an H3K9me3-independent, genomic shunt for regulating cell fates. Nucleic Acids Res. 2022; 50:7326–49. 10.1093/nar/gkac531. 35776115 PMC9303280

[25] Tan SL , Nishi M , Ohtsuka T , Matsui T , Takemoto K , Kamio-Miura A , Aburatani H , Shinkai Y , Kageyama R . Essential roles of the histone methyltransferase ESET in the epigenetic control of neural progenitor cells during development. Development. 2012; 139:3806–16. 10.1242/dev.082198. 22991445

[26] Minkovsky A , Sahakyan A , Rankin-Gee E , Bonora G , Patel S , Plath K . The Mbd1-Atf7ip-Setdb1 pathway contributes to the maintenance of X chromosome inactivation. Epigenetics Chromatin. 2014; 7:12. 10.1186/1756-8935-7-12. 25028596 PMC4099106

[27] Cruvinel E , Budinetz T , Germain N , Chamberlain S , Lalande M , Martins-Taylor K . Reactivation of maternal SNORD116 cluster via SETDB1 knockdown in Prader-Willi syndrome iPSCs. Hum Mol Genet. 2014; 23:4674–85. 10.1093/hmg/ddu187. 24760766 PMC4481691

[28] Xu Q , Goldstein J , Wang P , Gadi IK , Labreche H , Rehder C , Wang WP , McConkie A , Xu X , Jiang YH . Chromosomal microarray analysis in clinical evaluation of neurodevelopmental disorders-reporting a novel deletion of SETDB1 and illustration of counseling challenge. Pediatr Res. 2016; 80:371–81. 10.1038/pr.2016.101. 27119313 PMC5382808

[29] Karanth AV , Maniswami RR , Prashanth S , Govindaraj H , Padmavathy R , Jegatheesan SK , Mullangi R , Rajagopal S . Emerging role of SETDB1 as a therapeutic target. Expert Opin Ther Targets. 2017; 21:319–31. 10.1080/14728222.2017.1279604. 28076698

[30] Zhao W , Zhang S , Wang B , Huang J , Lu WW , Chen D . Runx2 and microRNA regulation in bone and cartilage diseases. Ann N Y Acad Sci. 2016; 1383:80–87. 10.1111/nyas.13206. 27526290 PMC5118124

[31] Yang L , Lawson KA , Teteak CJ , Zou J , Hacquebord J , Patterson D , Ghatan AC , Mei Q , Zielinska-Kwiatkowska A , Bain SD , Fernandes RJ , Chansky HA . ESET histone methyltransferase is essential to hypertrophic differentiation of growth plate chondrocytes and formation of epiphyseal plates. Dev Biol. 2013; 380:99–110. 10.1016/j.ydbio.2013.04.031. 23652029 PMC3885423

[32] Wan C , Zhang F , Yao H , Li H , Tuan RS . Histone Modifications and Chondrocyte Fate: Regulation and Therapeutic Implications. Front Cell Dev Biol. 2021; 9:626708. 10.3389/fcell.2021.626708. 33937229 PMC8085601

[33] Deng P , Chen QM , Hong C , Wang CY . Histone methyltransferases and demethylases: regulators in balancing osteogenic and adipogenic differentiation of mesenchymal stem cells. Int J Oral Sci. 2015; 7:197–204. 10.1038/ijos.2015.41. 26674421 PMC5153596

[34] Takada I , Kouzmenko AP , Kato S . Wnt and PPARgamma signaling in osteoblastogenesis and adipogenesis. Nat Rev Rheumatol. 2009; 5:442–47. 10.1038/nrrheum.2009.137. 19581903

[35] Zhang J , Matsumura Y , Kano Y , Yoshida A , Kawamura T , Hirakawa H , Inagaki T , Tanaka T , Kimura H , Yanagi S , Fukami K , Doi T , Osborne TF , et al. Ubiquitination-dependent and -independent repression of target genes by SETDB1 reveal a context-dependent role for its methyltransferase activity during adipogenesis. Genes Cells. 2021; 26:513–29. 10.1111/gtc.12868. 33971063

[36] Garcia P , Jarassier W , Brun C , Giordani L , Agostini F , Kung WH , Peccate C , Ravent J , Fall S , Petit V , Cheung TH , Ait-Si-Ali S , Le Grand F . Setdb1 protects genome integrity in murine muscle stem cells to allow for regenerative myogenesis and inflammation. Dev Cell. 2024; 59:2375–92.e8. 10.1016/j.devcel.2024.05.012. 38848717

[37] Ceol CJ , Houvras Y , Jane-Valbuena J , Bilodeau S , Orlando DA , Battisti V , Fritsch L , Lin WM , Hollmann TJ , Ferré F , Bourque C , Burke CJ , Turner L , et al. The histone methyltransferase SETDB1 is recurrently amplified in melanoma and accelerates its onset. Nature. 2011; 471:513–17. 10.1038/nature09806. 21430779 PMC3348545

[38] Zakharova VV , Magnitov MD , Del Maestro L , Ulianov SV , Glentis A , Uyanik B , Williart A , Karpukhina A , Demidov O , Joliot V , Vassetzky YS , Mège RM , Piel M , et al. SETDB1 fuels the lung cancer phenotype by modulating epigenome, 3D genome organization and chromatin mechanical properties. Nucleic Acids Res. 2022; 50:4389–13. 10.1093/nar/gkac234. 35474385 PMC9071401

[39] Ueshima S , Fang J . Histone H3K9 methyltransferase SETDB1 augments invadopodia formation to promote tumor metastasis. Oncogene. 2022; 41:3370–80. 10.1038/s41388-022-02345-3. 35546351 PMC9801494

[40] Olcina MM , Leszczynska KB , Senra JM , Isa NF , Harada H , Hammond EM . H3K9me3 facilitates hypoxia-induced p53-dependent apoptosis through repression of APAK. Oncogene. 2016; 35:793–99. 10.1038/onc.2015.134. 25961932 PMC4753255

[41] Yang H , Sui L , Cai C , Chu H , Diao Y . SETDB1 promotes progression through upregulation of SF3B4 expression and regulates the immunity in ovarian cancer. J Ovarian Res. 2024; 17:34. 10.1186/s13048-024-01358-8. 38317200 PMC10840244

[42] Cao N , Yu Y , Zhu H , Chen M , Chen P , Zhuo M , Mao Y , Li L , Zhao Q , Wu M , Ye M . SETDB1 promotes the progression of colorectal cancer via epigenetically silencing p21 expression. Cell Death Dis. 2020; 11:351. 10.1038/s41419-020-2561-6. 32393761 PMC7214465

[43] Regina C , Compagnone M , Peschiaroli A , Lena A , Annicchiarico-Petruzzelli M , Piro MC , Melino G , Candi E . Setdb1, a novel interactor of ΔNp63, is involved in breast tumorigenesis. Oncotarget. 2016; 7:28836–48. 10.18632/oncotarget.7089. 26840455 PMC5045360

[44] Yang W , Su Y , Hou C , Chen L , Zhou D , Ren K , Zhou Z , Zhang R , Liu X . SETDB1 induces epithelial-mesenchymal transition in breast carcinoma by directly binding with Snail promoter. Oncol Rep. 2019; 41:1284–92. 10.3892/or.2018.6871. 30483750

[45] Zhang H , Cai K , Wang J , Wang X , Cheng K , Shi F , Jiang L , Zhang Y , Dou J . MiR-7, inhibited indirectly by lincRNA HOTAIR, directly inhibits SETDB1 and reverses the EMT of breast cancer stem cells by downregulating the STAT3 pathway. Stem Cells. 2014; 32:2858–68. 10.1002/stem.1795. 25070049

[46] Qian X , Yang Y , Deng Y , Liu Y , Zhou Y , Han F , Xu Y , Yuan H . SETDB1 induces lenalidomide resistance in multiple myeloma cells via epithelial-mesenchymal transition and PI3K/AKT pathway activation. Exp Ther Med. 2023; 25:274. 10.3892/etm.2023.11973. 37206551 PMC10189757

[47] Klonou A , Korkolopoulou P , Giannopoulou AI , Kanakoglou DS , Pampalou A , Gargalionis AN , Sarantis P , Mitsios A , Sgouros S , Papavassiliou AG , Piperi C . Histone H3K9 methyltransferase SETDB1 overexpression correlates with pediatric high-grade gliomas progression and prognosis. J Mol Med (Berl). 2023; 101:387–401. 10.1007/s00109-023-02294-8. 36811655

[48] Na HH , Moon S , Kim KC . Knockout of SETDB1 gene using the CRISPR/cas-9 system increases migration and transforming activities via complex regulations of E-cadherin, β-catenin, STAT3, and Akt. Biochem Biophys Res Commun. 2020; 533:486–92. 10.1016/j.bbrc.2020.09.026. 32972752

[49] McEachron TA , Helman LJ . Recent Advances in Pediatric Cancer Research. Cancer Res. 2021; 81:5783–99. 10.1158/0008-5472.CAN-21-1191. 34561271 PMC9725930

[50] Mayers JG , Gokgoz N , Wunder JS , Andrulis IL . Abstract B39: Investigation of the effects of alterations in the glutamate receptor, GRIK2, on osteosarcoma tumorigenesis. Clin Cancer Res. 2018; 24:B39. 10.1158/1557-3265.SARCOMAS17-B39.

[51] Cuellar TL , Herzner AM , Zhang X , Goyal Y , Watanabe C , Friedman BA , Janakiraman V , Durinck S , Stinson J , Arnott D , Cheung TK , Chaudhuri S , Modrusan Z , et al. Silencing of retrotransposons by SETDB1 inhibits the interferon response in acute myeloid leukemia. J Cell Biol. 2017; 216:3535–49. 10.1083/jcb.201612160. 28887438 PMC5674883

[52] Johnson E , Salari K , Yang S . SETDB1: A perspective into immune cell function and cancer immunotherapy. Immunology. 2023; 169:3–12. 10.1111/imm.13619. 36524435 PMC10121739

[53] Johnson WE . Origins and evolutionary consequences of ancient endogenous retroviruses. Nat Rev Microbiol. 2019; 17:355–70. 10.1038/s41579-019-0189-2. 30962577

[54] Chuong EB , Elde NC , Feschotte C . Regulatory evolution of innate immunity through co-option of endogenous retroviruses. Science. 2016; 351:1083–87. 10.1126/science.aad5497. 26941318 PMC4887275

[55] Ukadike KC , Mustelin T . Implications of Endogenous Retroelements in the Etiopathogenesis of Systemic Lupus Erythematosus. J Clin Med. 2021; 10:856. 10.3390/jcm10040856. 33669709 PMC7922054

[56] Tovo PA , Galliano I , Parodi E , Calvi C , Gambarino S , Licciardi F , Dini M , Montanari P , Branca M , Ramenghi U , Bergallo M . Children with Chronic Immune Thrombocytopenia Exhibit High Expression of Human Endogenous Retroviruses TRIM28 and SETDB1. Genes (Basel). 2023; 14:1569. 10.3390/genes14081569. 37628621 PMC10454145

[57] Zhang SM , Cai WL , Liu X , Thakral D , Luo J , Chan LH , McGeary MK , Song E , Blenman KRM , Micevic G , Jessel S , Zhang Y , Yin M , et al. KDM5B promotes immune evasion by recruiting SETDB1 to silence retroelements. Nature. 2021; 598:682–87. 10.1038/s41586-021-03994-2. 34671158 PMC8555464

[58] Griffin GK , Wu J , Iracheta-Vellve A , Patti JC , Hsu J , Davis T , Dele-Oni D , Du PP , Halawi AG , Ishizuka JJ , Kim SY , Klaeger S , Knudsen NH , et al. Epigenetic silencing by SETDB1 suppresses tumour intrinsic immunogenicity. Nature. 2021; 595:309–14. 10.1038/s41586-021-03520-4. 33953401 PMC9166167

[59] Zhao Z , Feng L , Peng X , Ma T , Tong R , Zhong L . Role of histone methyltransferase SETDB1 in regulation of tumourigenesis and immune response. Front Pharmacol. 2022; 13:1073713. 10.3389/fphar.2022.1073713. 36582533 PMC9793902

[60] Takikita S , Muro R , Takai T , Otsubo T , Kawamura YI , Dohi T , Oda H , Kitajima M , Oshima K , Hattori M , Endo TA , Toyoda T , Weis J , et al. A Histone Methyltransferase ESET Is Critical for T Cell Development. J Immunol. 2016; 197:2269–79. 10.4049/jimmunol.1502486. 27511731

[61] Koirala P , Roth ME , Gill J , Piperdi S , Chinai JM , Geller DS , Hoang BH , Park A , Fremed MA , Zang X , Gorlick R . Immune infiltration and PD-L1 expression in the tumor microenvironment are prognostic in osteosarcoma. Sci Rep. 2016; 6:30093. 10.1038/srep30093. 27456063 PMC4960483

[62] Park JA , Cheung NV . Promise and Challenges of T Cell Immunotherapy for Osteosarcoma. Int J Mol Sci. 2023; 24:12520. 10.3390/ijms241512520. 37569894 PMC10419531

[63] Bielack SS , Smeland S , Whelan JS , Marina N , Jovic G , Hook JM , Krailo MD , Gebhardt M , Pápai Z , Meyer J , Nadel H , Randall RL , Deffenbaugh C , et al, and EURAMOS-1 investigators. Methotrexate, Doxorubicin, and Cisplatin (MAP) Plus Maintenance Pegylated Interferon Alfa-2b Versus MAP Alone in Patients With Resectable High-Grade Osteosarcoma and Good Histologic Response to Preoperative MAP: First Results of the EURAMOS-1 Good Response Randomized Controlled Trial. J Clin Oncol. 2015; 33:2279–87. 10.1200/JCO.2014.60.0734. 26033801 PMC4486345

[64] Noh HJ , Kim KA , Kim KC . p53 down-regulates SETDB1 gene expression during paclitaxel induced-cell death. Biochem Biophys Res Commun. 2014; 446:43–48. 10.1016/j.bbrc.2014.02.053. 24565839

[65] Ryu H , Lee J , Hagerty SW , Soh BY , McAlpin SE , Cormier KA , Smith KM , Ferrante RJ . ESET/SETDB1 gene expression and histone H3 (K9) trimethylation in Huntington’s disease. Proc Natl Acad Sci U S A. 2006; 103:19176–81. 10.1073/pnas.0606373103. 17142323 PMC1748195

[66] Lee JK , Kim KC . DZNep, inhibitor of S-adenosylhomocysteine hydrolase, down-regulates expression of SETDB1 H3K9me3 HMTase in human lung cancer cells. Biochem Biophys Res Commun. 2013; 438:647–52. 10.1016/j.bbrc.2013.07.128. 23933322

[67] Guo Y , Mao X , Xiong L , Xia A , You J , Lin G , Wu C , Huang L , Wang Y , Yang S . Structure-Guided Discovery of a Potent and Selective Cell-Active Inhibitor of SETDB1 Tudor Domain. Angew Chem Int Ed Engl. 2021; 60:8760–65. 10.1002/anie.202017200. 33511756

[68] Lee G , Elwood F , McNally J , Weiszmann J , Lindstrom M , Amaral K , Nakamura M , Miao S , Cao P , Learned RM , Chen JL , Li Y . T0070907, a selective ligand for peroxisome proliferator-activated receptor gamma, functions as an antagonist of biochemical and cellular activities. J Biol Chem. 2002; 277:19649–57. 10.1074/jbc.M200743200. 11877444

[69] Brust R , Lin H , Fuhrmann J , Asteian A , Kamenecka TM , Kojetin DJ . Modification of the Orthosteric PPARγ Covalent Antagonist Scaffold Yields an Improved Dual-Site Allosteric Inhibitor. ACS Chem Biol. 2017; 12:969–78. 10.1021/acschembio.6b01015. 28165718 PMC5652320

[70] Brust R , Shang J , Fuhrmann J , Mosure SA , Bass J , Cano A , Heidari Z , Chrisman IM , Nemetchek MD , Blayo AL , Griffin PR , Kamenecka TM , Hughes TS , Kojetin DJ . A structural mechanism for directing corepressor-selective inverse agonism of PPARγ. Nat Commun. 2018; 9:4687. 10.1038/s41467-018-07133-w. 30409975 PMC6224492

[71] Weichselbaum R , Little JB , Nove J . Response of human osteosarcoma *in vitro* to irradiation: evidence for unusual cellular repair activity. Int J Radiat Biol Relat Stud Phys Chem Med. 1977; 31:295–99. 10.1080/09553007714550351. 300732

[72] Dinçbaş FO , Koca S , Mandel NM , Hiz M , Dervişoğlu S , Seçmezacar H , Oksüz DC , Ceylaner B , Uzel B . The role of preoperative radiotherapy in nonmetastatic high-grade osteosarcoma of the extremities for limb-sparing surgery. Int J Radiat Oncol Biol Phys. 2005; 62:820–28. 10.1016/j.ijrobp.2004.11.006. 15936566

[73] Chiappinelli KB , Strissel PL , Desrichard A , Li H , Henke C , Akman B , Hein A , Rote NS , Cope LM , Snyder A , Makarov V , Budhu S , Slamon DJ , et al. Inhibiting DNA Methylation Causes an Interferon Response in Cancer via dsRNA Including Endogenous Retroviruses. Cell. 2015; 162:974–86. 10.1016/j.cell.2015.07.011. 26317466 PMC4556003

[74] Roulois D , Loo Yau H , Singhania R , Wang Y , Danesh A , Shen SY , Han H , Liang G , Jones PA , Pugh TJ , O’Brien C , De Carvalho DD . DNA-Demethylating Agents Target Colorectal Cancer Cells by Inducing Viral Mimicry by Endogenous Transcripts. Cell. 2015; 162:961–73. 10.1016/j.cell.2015.07.056. 26317465 PMC4843502

[75] Pan D , Bao X , Hu M , Jiao M , Li F , Li CY . SETDB1 Restrains Endogenous Retrovirus Expression and Antitumor Immunity during Radiotherapy. Cancer Res. 2022; 82:2748–60. 10.1158/0008-5472.CAN-21-3523. 35648422 PMC9357127

[76] Audinot B , Drubay D , Gaspar N , Mohr A , Cordero C , Marec-Bérard P , Lervat C , Piperno-Neumann S , Jimenez M , Mansuy L , Castex MP , Revon-Riviere G , Marie-Cardine A , et al. ctDNA quantification improves estimation of outcomes in patients with high-grade osteosarcoma: a translational study from the OS2006 trial. Ann Oncol. 2024; 35:559–68. 10.1016/j.annonc.2023.12.006. 38142939

